# EEG and Eye Tracking Demonstrate Vigilance Enhancement with Challenge Integration

**DOI:** 10.3389/fnhum.2016.00273

**Published:** 2016-06-07

**Authors:** Indu P. Bodala, Junhua Li, Nitish V. Thakor, Hasan Al-Nashash

**Affiliations:** ^1^Singapore Institute for Neurotechnology (SINAPSE), National University of SingaporeSingapore, Singapore; ^2^NUS Graduate School of Integrative Sciences and Engineering, National University of SingaporeSingapore, Singapore; ^3^Department of Electrical Engineering, College of Engineering, American University of SharjahSharjah, UAE

**Keywords:** vigilance enhancement, cognitive enhancement, EEG oscillations, relative band power, eye tracking, surveillance

## Abstract

Maintaining vigilance is possibly the first requirement for surveillance tasks where personnel are faced with monotonous yet intensive monitoring tasks. Decrement in vigilance in such situations could result in dangerous consequences such as accidents, loss of life and system failure. In this paper, we investigate the possibility to enhance vigilance or sustained attention using “challenge integration,” a strategy that integrates a primary task with challenging stimuli. A primary surveillance task (identifying an intruder in a simulated factory environment) and a challenge stimulus (periods of rain obscuring the surveillance scene) were employed to test the changes in vigilance levels. The effect of integrating challenging events (resulting from artificially simulated rain) into the task were compared to the initial monotonous phase. EEG and eye tracking data is collected and analyzed for *n* = 12 subjects. Frontal midline theta power and frontal theta to parietal alpha power ratio which are used as measures of engagement and attention allocation show an increase due to challenge integration (*p* < 0.05 in each case). Relative delta band power of EEG also shows statistically significant suppression on the frontoparietal and occipital cortices due to challenge integration (*p* < 0.05). Saccade amplitude, saccade velocity and blink rate obtained from eye tracking data exhibit statistically significant changes during the challenge phase of the experiment (*p* < 0.05 in each case). From the correlation analysis between the statistically significant measures of eye tracking and EEG, we infer that saccade amplitude and saccade velocity decrease with vigilance decrement along with frontal midline theta and frontal theta to parietal alpha ratio. Conversely, blink rate and relative delta power increase with vigilance decrement. However, these measures exhibit a reverse trend when challenge stimulus appears in the task suggesting vigilance enhancement. Moreover, the mean reaction time is lower for the challenge integrated phase (*RTmean* = 3.65 ± 1.4*s*) compared to initial monotonous phase without challenge (*RTmean* = 4.6 ± 2.7*s*). Our work shows that vigilance level, as assessed by response of these vital signs, is enhanced by challenge integration.

## 1. Introduction

The term “cognitive enhancement” can be thought of as genetic, neuropharmaceutical, computer or direct neural interventions to extend the abilities of the brain (Bostrom and Sandberg, 2009). Literature contains a large number of conventional means for cognitive enhancement including education, mental training, encoding strategies, meditation, yoga, tai chi, martial arts, sports and exercise, caffeine, nicotine, diet and herbal extracts (Vaynman and Gomez-Pinilla, 2005; Bostrom and Sandberg, 2009; Lambourne and Tomporowski, 2010; Nilsson et al., 2012; Converse et al., 2014). There have been reports of other nonconventional, more contemporary means of cognitive enhancers including pharmaceuticals, psychological interventions, molecular and gene therapy, transcranial magnetic stimulation, transcranial direct current stimulation and neural implants (Lynch, 2002; Fregni et al., 2005; Shirvalkar et al., 2006; Hallett, 2007; Sahakian and Morein-Zamir, 2007; Lucke et al., 2011). Recently several studies have investigated non invasive and non pharmacological approaches like training of certain cognitive abilities to enhance cognitive performance of individuals (Bamidis et al., 2014; Robert et al., 2014). For example, the use of computer games has been reported to play a significant role in cognitive enhancement (Green and Bavelier, 2003; Owen et al., 2010; Anguera et al., 2013). Recently, Anguera et al. (2013) demonstrated that computer gaming can significantly improve cognitive control in older adults. Similar studies were conducted to enhance specific functions like visuomotor skills, working memory and attention (Klingberg et al., 2005; Jaeggi et al., 2008; Strenziok et al., 2014).

In this research, we tackle the challenge of enhancing cognitive performance by maintaining vigilance of a subject performing long duration monotonous tasks such as surveillance. Monitoring or surveillance activities require the personnel to maintain vigilance over long periods of time to detect the target events which occur rarely, if at all. However the ability to sustain vigilance is limited and is affected by several factors like nature of stimuli, time of the day and stress levels (Sarter et al., 2001). It has been well demonstrated that long duration search tasks are often accompanied by increase in reaction time and sometimes by decrease in the rate of target detection (Matthews and Davies, 2001; Pattyn et al., 2008; Head and Helton, 2012). Pattyn et al. (2008) have shown that target detection performance decreases by 15% in 30 min of a monotonous task. Oken et al. (2006) have explored the physiological basis of vigilance and have established that the neural mechanisms and multiple constructs underlying vigilance are not unidimensional. Earlier studies of vigilance decrement were broadly based on two theories. Some studies suggest that vigilance decrement is because of the increase in task-unrelated thoughts which suggest withdrawal of attention system due to insufficient mental workload often known as “underload” hypothesis (Smallwood et al., 2004; Pattyn et al., 2008; Braboszcz and Delorme, 2011). There are also studies which suggested that the decrement in vigilance is due to depletion of cognitive resources as a result of accumulated fatigue over time referred to as “overload” hypothesis (Warm et al., 2008; Helton and Russell, 2010, 2015). However, none of the theories completely explain vigilance decrement. Recent studies propose that a combination of both overload and underload theories could explain vigilance decrement (Langner et al., 2010, 2012; Thomson et al., 2015).

Neurological signals such as electroencephalography (EEG) and physiological measures such as eye tracking and heart rate variability can be used to predict the engagement and vigilance decrement during various tasks (Berka et al., 2007; Martel et al., 2014). Eye tracking technology is being increasingly employed in the study of cognitive performance in naturalistic conditions. Eye movements have been shown to predict changes in the neural activity with high accuracy facilitating the researchers to study several cognitive phenomena in a highly non-invasive fashion. For example, Di Stasi et al. (2013) concluded that saccade velocity can be used reliably to measure the changes in the arousal level. Eye blink frequency and duration are used to estimate vigilance levels where both the measures increased as the performance declined in a vigilance task (McIntire et al., 2014b). Blink frequency has also been studied in Yamada (1998) along with frontal theta power of EEG where the authors found that an interesting task led to inhibition of the eye blinks. In Gao et al. (2015), the authors used percentage of eye closure (PERCLOS) over time obtained from eye tracking to detect the onset of fatigue during driving. Several other measures like pupil size, pupil velocity, eye movements durations were also used to develop performance monitors during vigilance tasks (McIntire et al., 2014a). However, the findings from these studies still require deeper understanding of vigilance mechanism and its correlation with the measures obtained from the eye tracking data.

Several studies investigated the correlations between the frequency and amplitude changes in EEG and behavioral performance on tasks related to sustained attention to determine markers for onset of vigilance decrement (Matousek and Petersén, 1983; Makeig and Inlow, 1993; Gevins et al., 1997). Researchers have developed indices derived from the power spectral variables where individual band power changes and the ratios between them served as markers for drop in engagement during sustained attention tasks. Engagement Index (EI), the ratio of beta to the sum of alpha and theta, has been developed and used in several neurofeedback based BCI studies for timely estimation of changes in arousal (Scerbo et al., 2003; Freeman et al., 2004; Berka et al., 2007). Another index which is in use is the ratio of frontal theta power to parietal alpha power (Gevins et al., 1997; Smith and Gevins, 2005). The results from these studies showed an increased frontal theta response along with reduced parietal alpha during demanding tasks where an increase in allocation of attention is observed. This index has also been used to determine engagement and cortical activation. Frontal midline theta also serves as a good indicator for attention and concentration (Yamada, 1998; Mitchell et al., 2008). In Yamada (1998), the authors concluded that a contextually interesting task (playing video games compared to watching a boring animation) seems to provoke an increased activity of theta band in the midline frontal electrodes and inhibit eye blinks. While these indices serve as indicators for subjects' engagement, delta band power is known to indicate the amount of fatigue in a long duration driving task (Lal and Craig, 2001; Chuang et al., 2014). It was found that delta activity increases during transition to drowsiness and during sleep. Also, in Chuang et al. (2014), the authors found that delta activity decreased during optimal performance state induced by kinesthetic feedback in a driving task. In addition to amplitude of band powers, spatial changes in frequency bands have been studied by Clayton et al. (2015) to provide more accurate estimation of changes in vigilance levels and thereby to understand attention-related brain networks. For example, theta changes were prominently found in frontal regions whereas alpha changes were found all over the scalp (Strijkstra et al., 2003; Wascher et al., 2014). Detection of vigilance decrement has been also made possible using event related potentials (ERP). Long latency ERP components such as P300 suggested drop in vigilance (Smith et al., 2002). Decrease in amplitudes of ERP components with vigilance decrement were also reported (Kam et al., 2013; Staub et al., 2014). Efforts have been made by researchers to understand vigilance and develop strategies to reduce vigilance decrement with the intention of enhancing the level of task performance in long duration activities that require sustained attention. Neurofeedback training using EEG and fMRI based measures is usually employed as a strategy to train individuals to reduce inattention and mind-wandering. For example, deBettencourt et al. (2015) studied the possibility of training sustained attention through neurofeedback using real-time fMRI signals. Neurofeedback attention training is also used in Wang et al. (2015) to train persons with autism spectrum disorder to improve self regulation skills.

In this study, we hypothesize that vigilance enhancement can be achieved by using “challenge integration,” a strategy of integrating challenging stimuli with the primary task. Challenge increases cognitive workload during the task and helps alleviate vigilance decrement according to the “underload” hypothesis discussed earlier. A monitoring task in an industrial environment was designed. The subjects were asked to identify a target amongst distractors. We used noisy visual stimulus in the form of artificially simulated rain to act as challeging stimulus during monotonous visual search. In our previous studies, we found that noisy visual stimulus will increase mental workload (Bodala et al., 2014; Yu et al., 2015). Hence, we used noisy visual stimulus (rain) to create challenge in this study. EEG and eye tracking data were collected and analyzed to study the changes in vigilance levels due to challenge integration. The details of the experiment task and data collection are discussed in the next section. In Section 3, the results obtained from the analysis of EEG and eye tracking data were summarized and correlation analyses between significant EEG and eye tracking measures were performed. In addition, the eye tracking data obtained from a small cohort of 4 subjects was analyzed to compare the changes between sessions with and without challenge integration. The findings regarding the effect of challenge integration on the task and future directions are discussed in detail in Section 4 followed by concluding remarks in Section 5.

## 2. Materials and methods

### 2.1. Subjects

Twelve healthy students (9 females) of age 20–31 years (*mean* = 21.5 *years* and *std* = 1.68 *years*), with normal or corrected-to-normal vision, who have no previous nervous or psychiatric disorders and were not on medications were recruited from the National University of Singapore (NUS) to participate in the study. Four additional subjects (1 female) with similar demographics were recruited to participate in the control study. The study was approved by the Institutional Review Board (IRB) of the National University of Singapore. Written informed consent was obtained from each participant before the beginning of the experiment. All participants received monetary compensation for their time upon completion of the experiment.

### 2.2. Experimental setup

**Figure 1:**
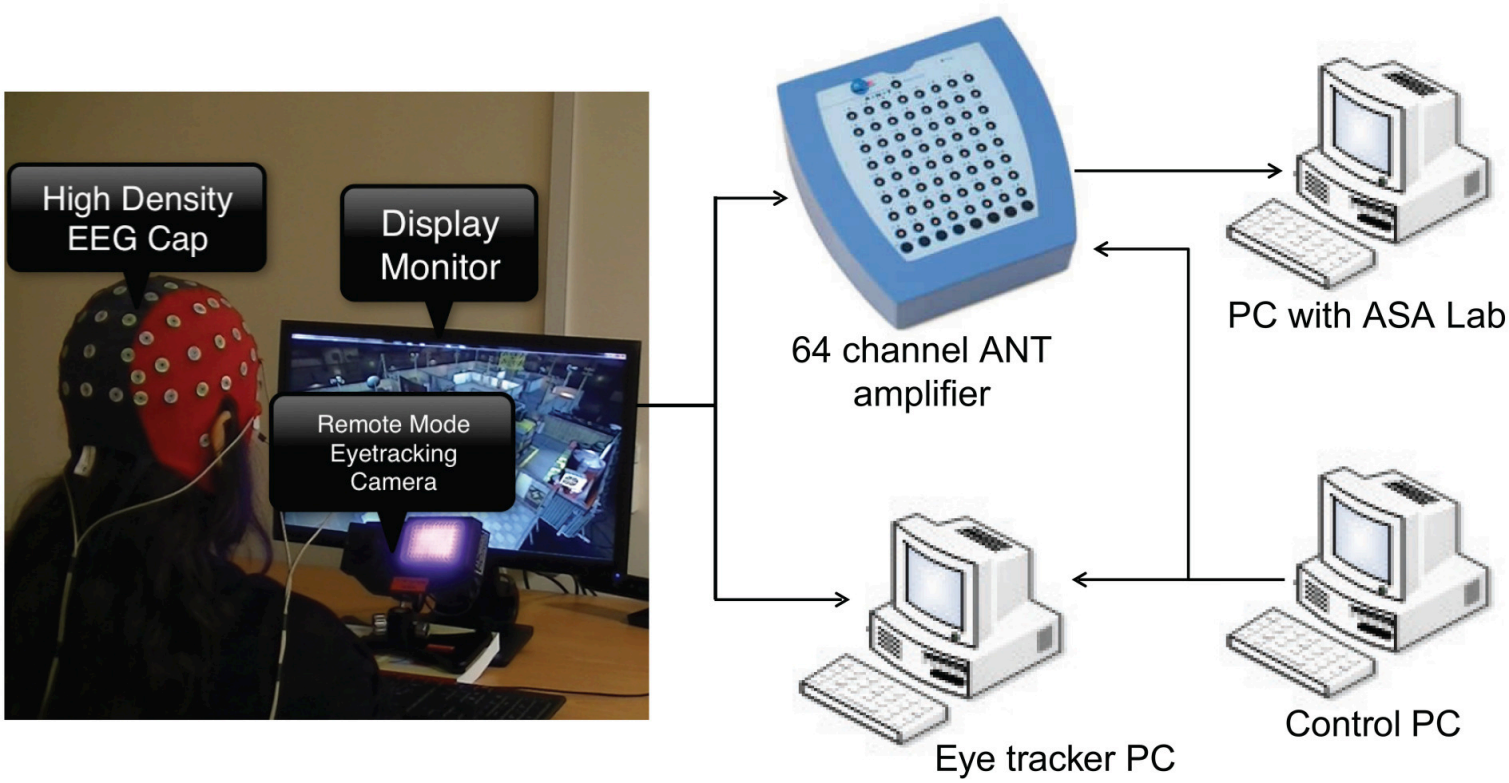
**The experimental setup showing EEG and eye tracking data acquisition**. Subject wearing EEG cap is seated in front of stimulus monitor and eye tracking camera.

The experimental setup is shown in Figure 1. Asalab™ data acquisition software, version 4.7.12 (ANT Neuro, Netherlands, www.ant-neuro.com) was used to record EEG data. Waveguard™ caps (CA-142; ANT Neuro, Netherlands, www.ant-neuro.com) with 64 electrodes placed in the international 10–10 system were used; sintered Ag/AgCl electrodes with 10*kΩ* resistor built in to prevent accidental heating in case of loops in the wires. The caps are also actively shielded to facilitate high quality EEG data recording by preventing 50/60 Hz environmental noise and artifacts arising from the movement of the cables. Extra electrodes were used to collect electrocardiogram (ECG) and horizontal and vertical electrooculogram (hEOG and vEOG). Eye tracking data was collected from the left eye of all the subjects using the Eyelink 1000 system in remote mode (SR Research, http://www.sr-research.com/), which means that no head/chin rest was used to stabilize subject's head. Nonetheless, the subjects were instructed not to make any drastic head movements. A marker was placed on the forehead of the subjects to correct for the changes in head position automatically by the tracker. Dell PC with 21″ screen was used as a stimulus monitor. The monitoring scene was presented to the subject on the stimulus monitor. The control PC with MATLAB and Psychtoolbox was used for running the codes to synchronize data collection and send event markers to eye tracker and EEG amplifier using a parallel port connection. The experiment was carried out in a quiet room with controlled level of luminance.

### 2.3. Procedure and task

Before the experiment, the subjects underwent a color blindness test. In addition, each subject completed other probing tests like the Epworth sleepiness scale test (ESS) (Johns, 1991) to ensure that none of the subjects were sleep deprived (*mean_score* = 5.33 ± 2.64). After signing the consent form, the subject was seated such that the distance between the eyes and the monitor is approximately 50 cm corresponding to a visual angle of 40 × 30°. Before the start of the experiment, the eye tracking system was calibrated. Each experiment lasted for approximately 90 min including subject preparation and the task with simultaneous data collection.

#### 2.3.1. Protocol

**Figure 2:**
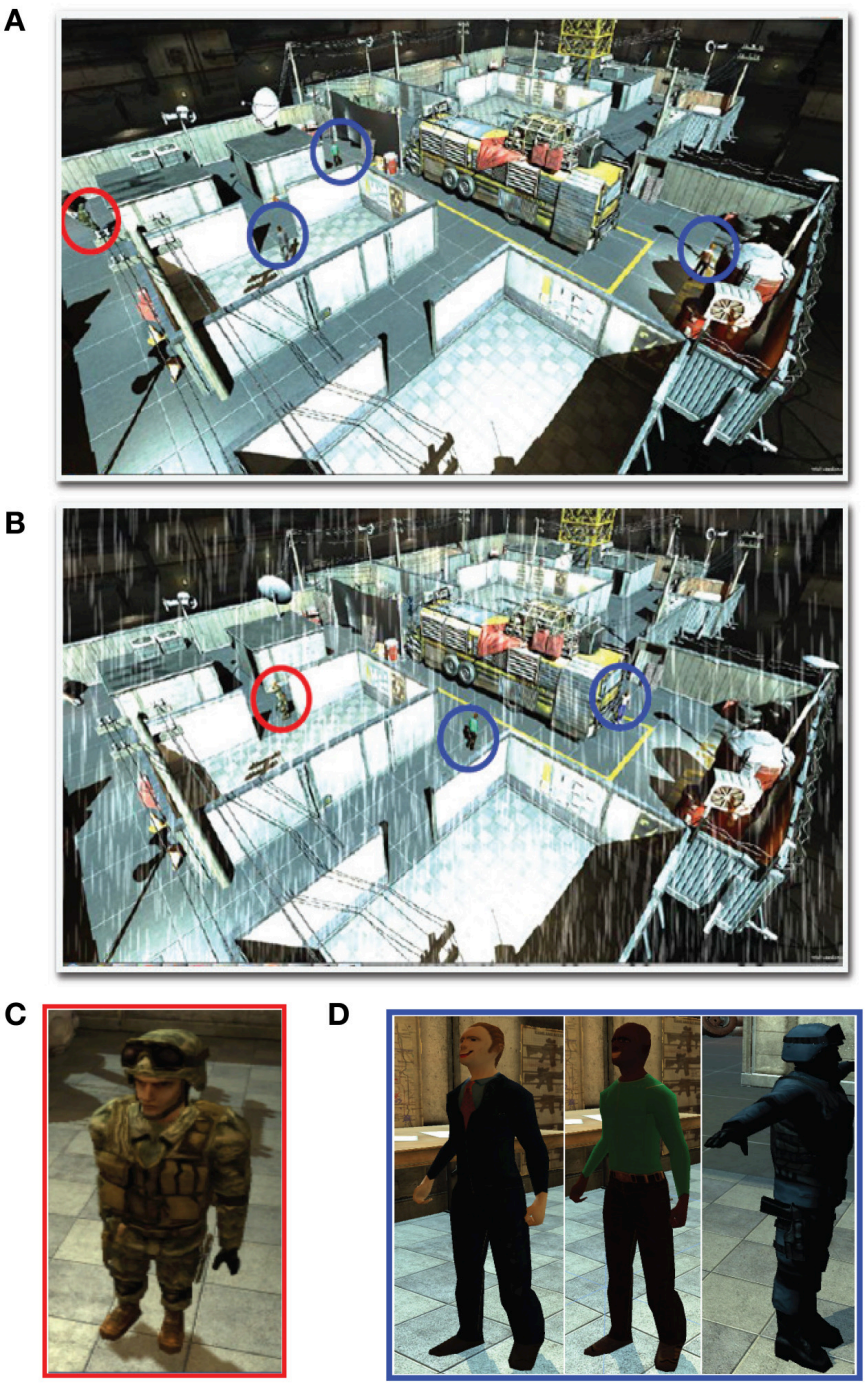
**Simulated industrial plant or warehouse (A) without challenge (rain) and (B) with challenge (rain) showing the (C) target intruder [circled red in (A,B)] and (D) distractors [circled blue in (A,B)]**.

The experiment paradigm was designed to test our hypothesis regarding challenge integration with the actual monitoring function. In this experiment, the target was selected to be an intruder wearing a military uniform (Figure 2C) appearing in different locations in an industrial plant or warehouse. At the beginning, the subject received sufficient training (~2–3 min) to differentiate between the target and the distractors (Figure 2D). The subject was then asked to watch various activities on the screen and hit letter Q whenever the target appeared on the screen. The sequence of events in this experiment was divided into three phases. In phase 1, the target appeared at an interval of once in 60–90 s at 20 places in the scene in a random manner (Figure 2A). This phase lasted for 15 min and was intended to trigger vigilance decrement. Then phase 2 was activated, where surprising occurrence of challenge (rain) commenced as shown in Figure 2B. Rain is expected to make target detection more challenging and requires additional cognitive effort. Phase 2 can be divided into four intervals of 5 min each with the first 3 min with rain (challenge) and the last 2 min without rain. The target appearance rate was maintained as in phase 1. The final phase of the experiment was similar to phase 1, a scene without challenge with target appearance at the rate of once in every 60–90 s for 10 min.

#### 2.3.2. Control study

We collected data from four subjects who underwent the experiment twice—with and without challenging stimulus in phase 2. This was intended to compare the effect of challenge integration on the whole task. Two of the subjects (randomly selected) performed the experiment with challenge first while the other two performed the experiment without challenge first. Saccade velocity of these subjects is analyzed and compared for the sessions with and without challenge. The results of the control study are detailed in Section 3.4.

### 2.4. Data processing

#### 2.4.1. Eye tracking data

The eye tracking data comprised of samples with time stamp, pupil size, eye position and eye velocity recorded at a sampling rate of 500 Hz. These values are stored as “sample lines” in the raw data file. The Eyelink system parses data samples into meaningful states (saccades, fixation, and blinks). Velocity and acceleration based algorithms are used by the tracker to detect saccades, the movement of the eye from one point to another on the screen. Based on previously set thresholds (*velocity*_*threshold* = 30°∕*s* and *acceleration*_*threshold* = 8000°∕*s*^2^), saccades are differentiated from fixations which are defined as the relatively stable positioning of the eyes on a specific point on the screen. Fixations are necessary to grasp the visual content at a specific point in the scene while saccades are necessary to understand the content of the scene and to select task relevant regions to attend to. Eye blinks are also detected by the eye tracker. The raw data file in .edf format is converted to .asc format which consists of “sample lines” and “event lines,” where the samples are categorized into different types of eye movements such as fixations, saccades and blinks by the tracker. We used Matlab to extract various measures related to different types of eye movements between the required intervals. A moving average filter of 60 s was used to obtain an average of these measures across time. The measures are then normalized within each subject before averaging them across all the subjects.

#### 2.4.2. EEG data

In our experiment with monitoring task, artifacts related to eye movements are not avoidable and needed to be removed. We employed least mean squares (LMS) algorithm to remove the influence of eye movements from EEG based on reference to the two EOG channels: horizontal EOG and vertical EOG (He et al., 2004). The LMS method of artifact removal is automatic and stable on long duration EEG recordings as present in this study. However, for techniques with lower computational costs and more robust artifact removal, readers may also refer to Puthusserypady and Ratnarajah (2005) and Plöchl et al. (2012). Then, canonical correlation analysis (CCA) was utilized to remove muscular artifacts by projecting EEG onto a few maximally auto-correlated components (De Clercq et al., 2006). All channels were then referenced to the values averaged over all channels at each time point. Short time Fourier transform (STFT) was used to convert time series data into spectral power representation. Non-overlapping time window of length of 10 s was used for STFT.

Let *P*_*i, j*_ be the spectral power at time point *i* and frequency *j*. Each band power can be calculated by

(1) $$\begin{array}{l} BP=\frac{1}{N}\overset{t_{N}}{\underset{i=t_{1}}{\sum }}\overset{f_{m2}}{\underset{j=f_{m1}}{\sum }}P_{i,j}. \end{array}$$

Where *t*_1_ and *t*_*N*_ are the starting and ending time points of the partitioned data, respectively. *f*_*m*1_ and *f*_*m*2_ are boundary frequencies of the desired power band, respectively. Total power is obtained by summing up the five band powers of interest (δ(0.5–4 Hz), θ (4–7 Hz), α (8–12 Hz), β (13–30 Hz), γ (31–45 Hz)) as follows

(2) $$\begin{array}{l} P_{total}=\underset{band\in \left\{\delta ,\theta ,\alpha ,\beta ,\gamma \right\}}{\sum }BP_{band}. \end{array}$$

Relative band power is defined as the ratio of that band power to the total power

(3) $$\begin{array}{l} P_{relative}=\frac{BP}{P_{total}}. \end{array}$$

## 3. Results

In this section we present the results from the analysis of EEG and eye tracking data and compare the measures obtained from the data across different phases of the experiment. The mean reaction time of phase 2 with integrated challenge (*RTmean* = 3.65 ± 1.4*s*) is found to be lower than phase 1 without challenge (*RTmean* = 4.6 ± 2.7*s*).

### 3.1. Eye tracking

**Figure 3:**
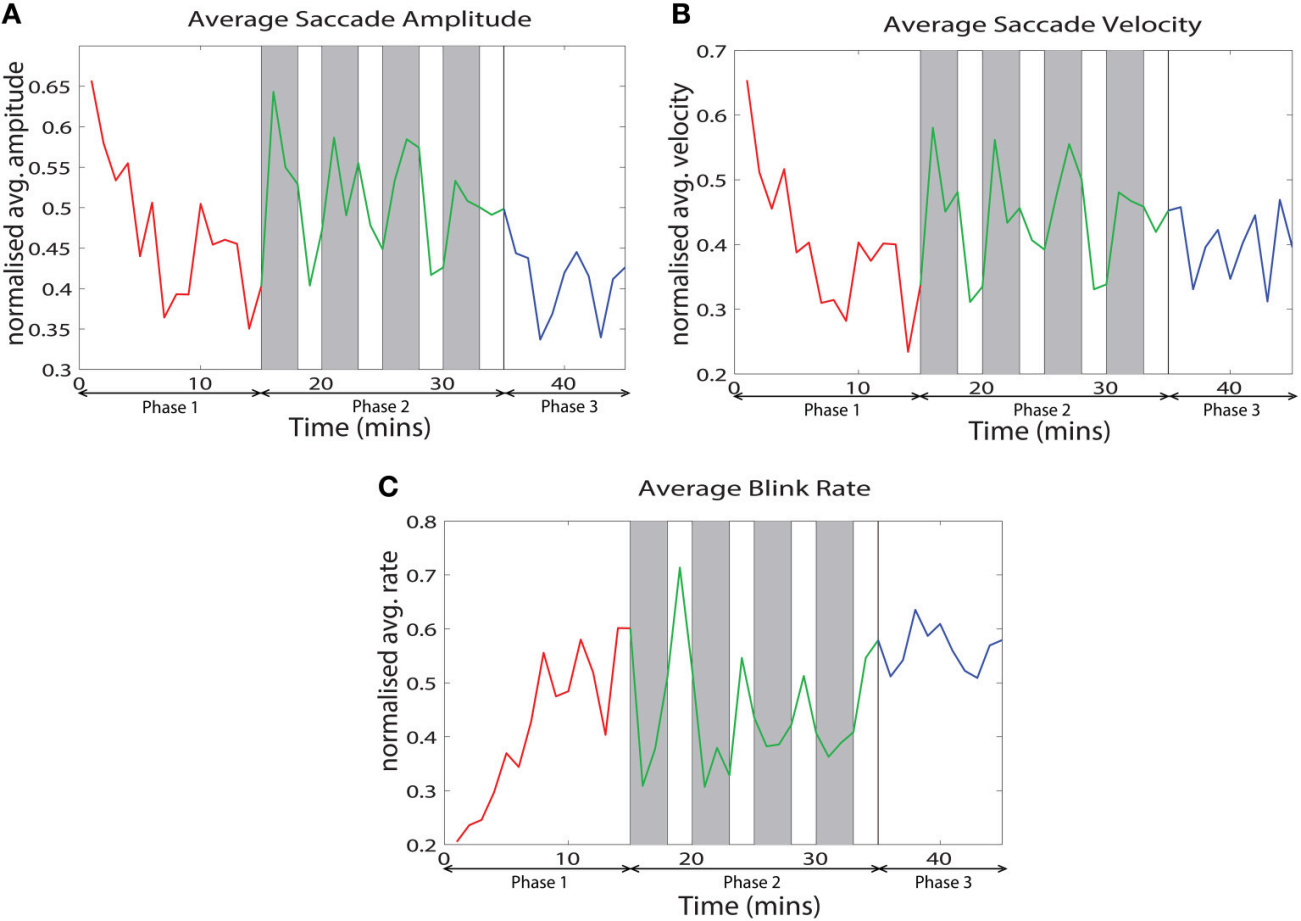
**Variation of the normalized average of (A) saccade amplitude, (B) saccade velocity and (C) blink frequency with time across all the subjects**.

We analyzed various eye tracking measures related to fixations, saccades and blinks to estimate the vigilance levels at different phases. The ANOVA analysis was performed across the last 10 min of phase 1, challenge period of phase 2 and phase 3 for the normalized average measures. We wanted to compare the vigilance enhancement level during the challenge phase with vigilance decrement period in phase 1. Since vigilance decrement is not significant in the first 5 min of the experiment we excluded this period from the ANOVA analysis. Table 1 shows eye tracking measures that exhibit statistically significant (*p* < 0.05) changes between the three phases of the experiment. Variation of these measures with time are shown in Figure 3. The saccade amplitude and velocity are found to decrease with time whereas blink rate increases with time during monotonous target detection in phase 1. In phase 2, rising peaks are observed during the challenge stimulation in the case of saccade amplitude and saccade velocity while blink rate falls during challenge stimulation. However in phase 3, these are maintained relatively constant.

**Table 1:** **ANOVA results for various eye tracking measures**.

**Dependent variable**	**η^2^**	***F***	***p***
Saccade velocity	0.197	4.48	0.0178
Saccade amplitude	0.188	4.33	0.0201
Blink frequency	0.207	4.34	0.0199

### 3.2. EEG

We analyzed EEG band power indices related to vigilance decrement that have been well accepted in the literature to estimate vigilance changes in our experiment.

**Figure 4:**
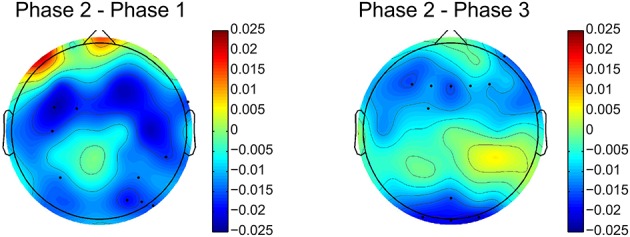
**Relative delta power differences between phase 2 and phase 1 and phase 2 and phase 3**. Black dots indicates electrodes with significant difference (*p* < 0.05, after FDR correction). Blue and red colors on difference topographies stand for the lower and higher powers in phase 2 respectively.

**Figure 5:**
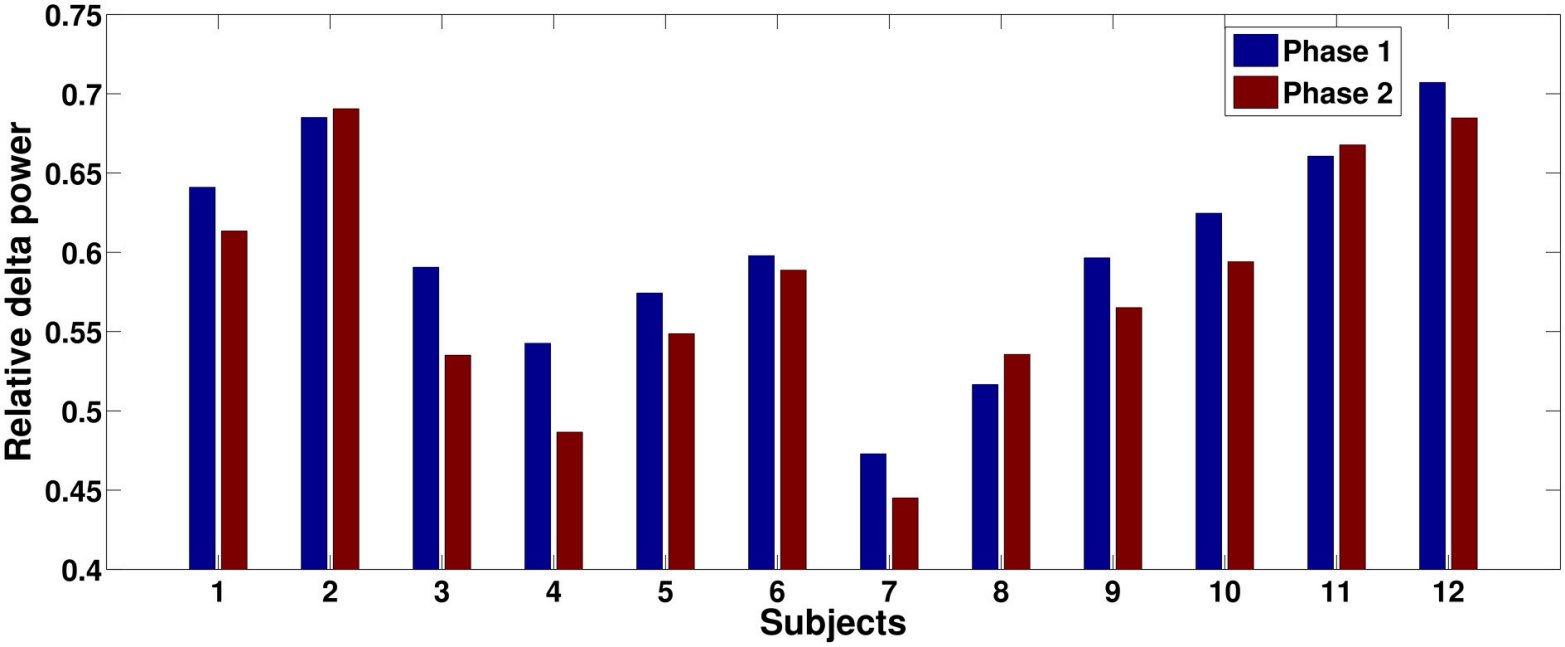
**Relative delta power comparisons between phase 1 and phase 2 for each subject on an illustrative channel, FC3**. Majority of subjects (9 out of 12) present lower relative delta power at phase 2.

The relative delta power is averaged across all subjects for the three phases. The relative power differences between phase 2 and other two phases are shown in Figure 4. The electrodes with significant differences (*p* < 0.05, after FDR correction) between phases are marked by black dots on the topographies. Figure 4 clearly shows that relative delta power on the frontal (extended to the parietal cortex) and occipital cortices decreased for the challenging condition (phase 2) compared to monotonous conditions (phase 1 and phase 3). The relative delta power during phase 2 and phase 1 on an illustrative channel, FC3, for each subject are listed in Figure 5. Majority of subjects (9 out of 12) present lower relative delta power at phase 2 (*p* < 0.01, Wilcoxon signed-rank test).

**Figure 6:**
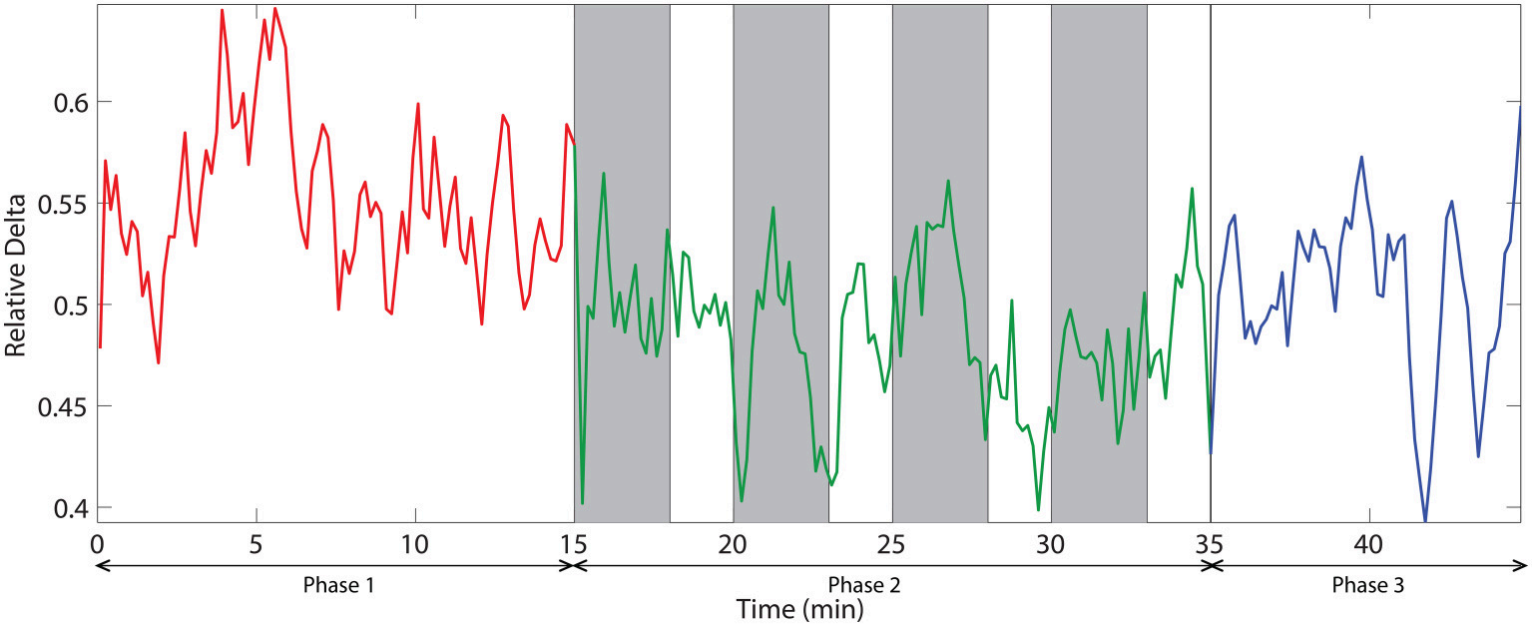
**The dynamic of relative delta power on an illustrative channel, FC3**. Red, green, and blue colors correspond to the phases 1, 2, and 3 respectively. Vertical gray bars indicate the periods with challenging events.

The dynamic of relative delta power for an illustrative channel, FC3 is shown in Figure 6. The changes in relative delta power with time clearly exhibit that challenging events modulate the power. At the beginning, the power was relatively low and then it increased with time. When challenging events were presented, the power was suppressed and kept at a relatively low level for almost all the time during phase 2. In the phase 3, the power rebounded to the same level as of the end of phase 1.

**Figure 7:**
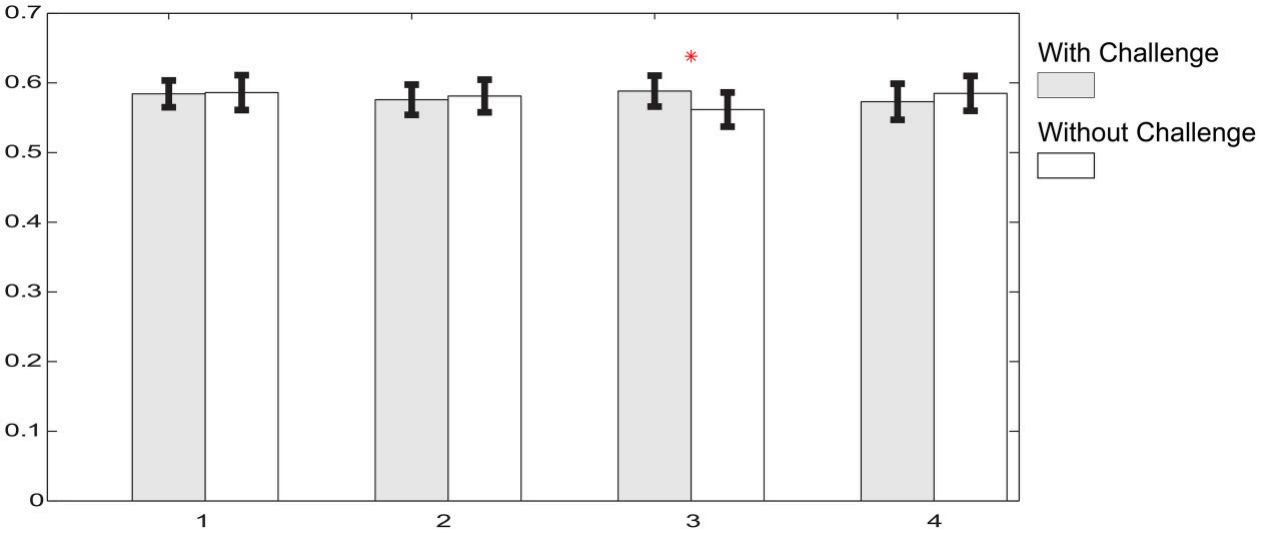
**Relative delta power comparisons between portions with and without challenge on a typical channel, FC3 in the phase 2**. Gray bars illustrate the relative delta power averaged within corresponding challenge periods, while white bars are for portions without challenge. Shape “I” at the top of each bar represents standard error. Asterisk above bars illustrates that there is significant power difference between periods with challenge and without challenge (*p* < 0.05).

To compare the effect of momentarily removing the challenge from the scene, we performed pairwise comparisons of the relative delta power between the four pairs of periods with and without challenge within phase 2. Phase 2 is divided into four 5 min periods where each 5 min period is comprised of 3 min with challenge and 2 min without challenge. Figure 7 depicts the relative delta power in each portion on a illustrative channel, FC3, which exhibited significant difference in the comparison between phase 2 and phase 1. Figure 7 shows that 3 out of 4 pairwise comparisons between periods with challenge and without challenge are not statistically significant. This suggest that there are no significant changes in the relative delta power even when the challenge is momentarily withdrawn. Also, portions with or without challenge do not exhibit any monotonic changes with time which shows that the effect of integrating challenge can be sustained during phase 2.

**Figure 8:**
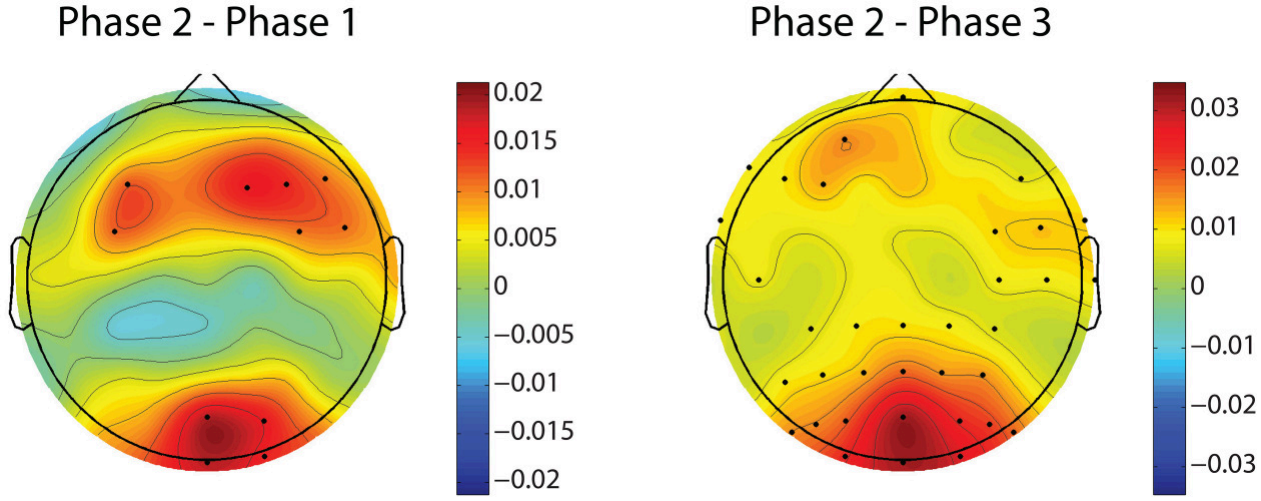
**Difference topographies of grand relative theta power averaged across subjects**. The left column shows difference topographies between phase 2 and phase 1, while the right column is for between phase 2 and phase 3. Black dots indicate electrodes with significant difference (*p* < 0.05, after FDR correction). Blue and red colors on difference topographies stand for the lower and higher powers in phase 2 respectively.

**Figure 9:**
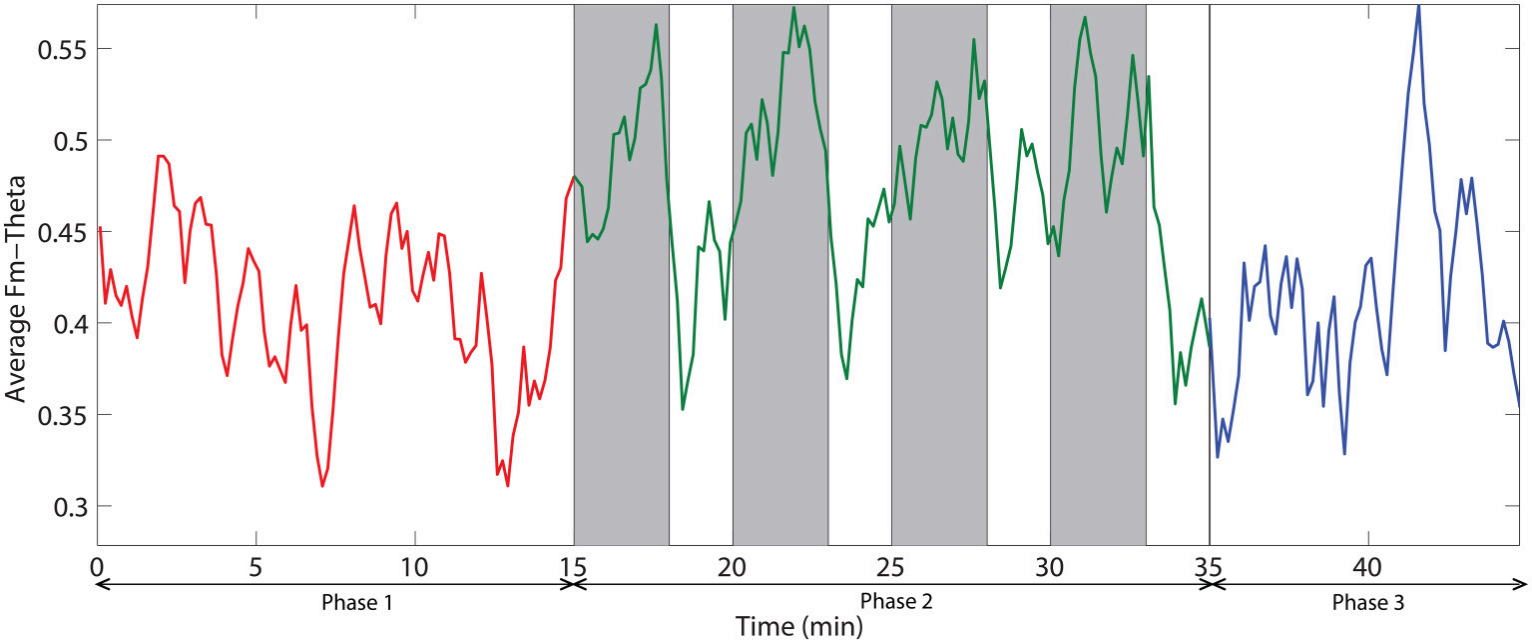
**The dynamic of frontal midline theta averaged across all subjects**. Red, green, and blue colors correspond to the phases 1, 2, and 3 respectively. Vertical gray bars indicate the periods with challenging events.

Figure 8 demonstrates the spatial changes in theta band power. Cortices with significant difference with respect to relative theta power are frontal and occipital regions. However, the significantly different occipital area is smaller and frontal area is larger in the case of the difference topography between phase 2 and phase 1 (*Phase*2–*Phase*1) compared to the difference topography between phase 2 and phase 3 (*Phase*2–*Phase*3). In addition, there is an obvious augmentation in phase 2 compared to phase 3 on the temporal cortex. The time dynamic of average relative theta power along the frontal midline electrodes (F3, F2, F4, F6, FC3, FC4, FC6) is plotted in Figure 9. There is a significant difference between the three phases as measured by ANOVA analysis (*p* < 0.05). Frontal midline theta decreased during the initial monotonous phase and increased significantly during challenge integrated periods.

**Figure 10:**
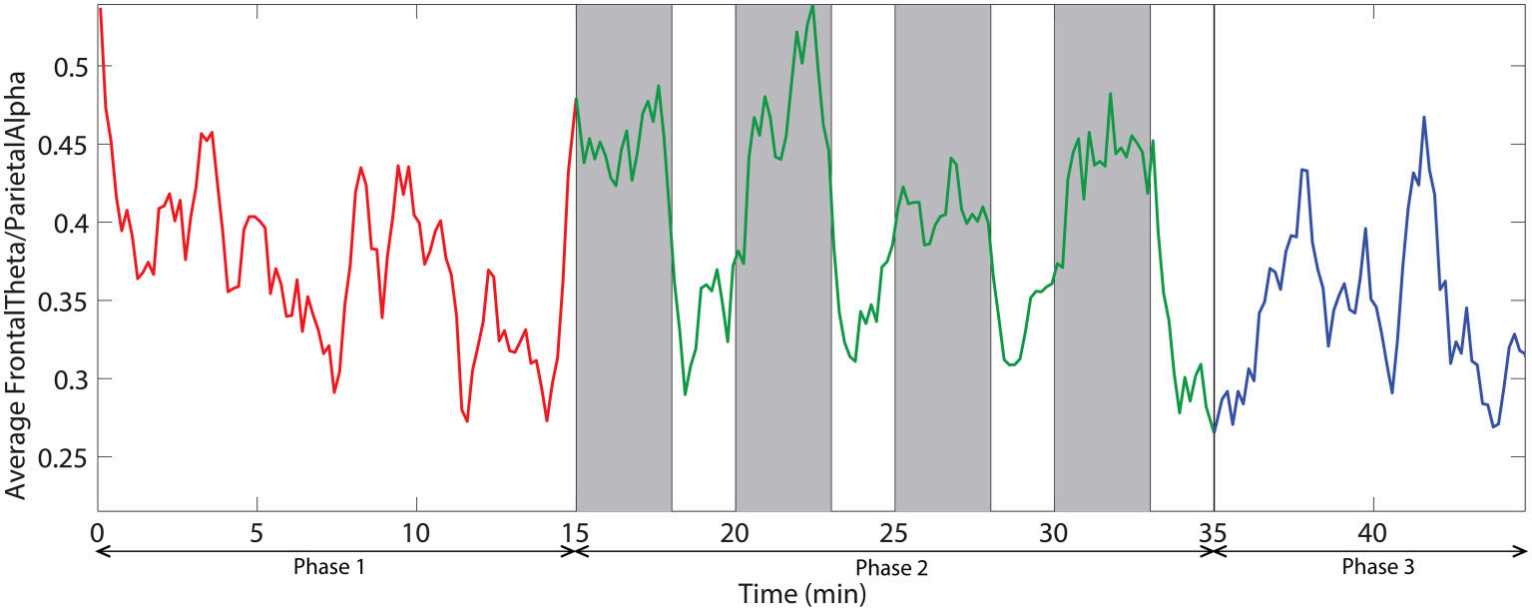
**The dynamic of frontal theta to parietal alpha ratio averaged across all subjects**. Red, green, and blue colors correspond to the phases 1, 2, and 3 respectively. Vertical gray bars indicate the periods with challenging events.

We also investigated the ratio of frontal theta power (F3, F2, F4, F6, FC3, FC4, FC6) to parietal alpha power (P3, P4, P5, P6, P7, P8). Figure 10 demonstrates the time dynamic of the frontal theta to parietal alpha ratio averaged across all subjects. ANOVA analysis on mean frontal theta to parietal alpha ratio across the three phases demonstrate with statistical significance (*p* < 0.05) that the ratio is higher for challenge integrated phase compared to monotonous phase.

### 3.3. Correlation between EEG and eye tracking data

**Figure 11:**
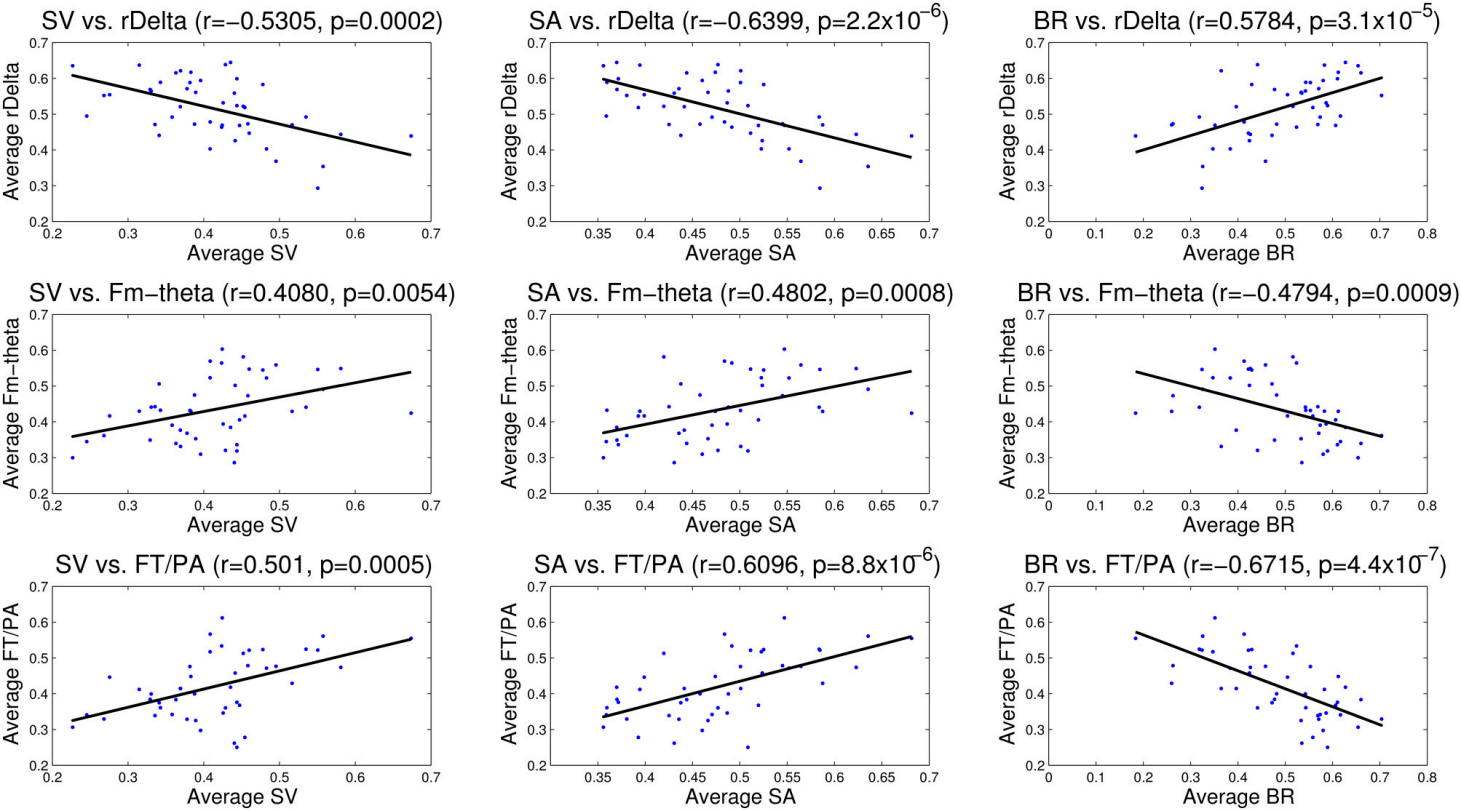
**Correlation analysis between EEG (relative delta power, frontal midline theta and frontal theta to parietal alpha ratio) and eye tracking (saccade velocity, saccade amplitude and blink rate) measures**. The correlation coefficient (*r*) and significance value (*p*) are also shown in each case. All the correlations are statistically significant.

The correlation analysis between EEG measures—relative delta power, frontal midline theta and frontal theta to parietal alpha ratio and the eye tracking measures—saccade velocity, saccade amplitude, and blink rate are shown in Figure 11. We found that saccade amplitude and saccade velocity are negatively correlated with relative delta power whereas blink rate is positively correlated. Conversely, saccade amplitude and saccade velocity are positively correlated with frontal midline theta and frontal theta to parietal alpha ratio whereas blink rate is negatively correlated. These results suggest that saccade measures (saccade amplitude and saccade velocity) of eye tracking and frontal midline theta and frontal theta to parietal alpha ratio of EEG correlate positively with vigilance level while blink rate from eye tracking and relative delta power from EEG correlate negatively with vigilance level. The correlations are similar to the observations made by the studies described in the literature in Section 1.

### 3.4. Control study

**Figure 12:**
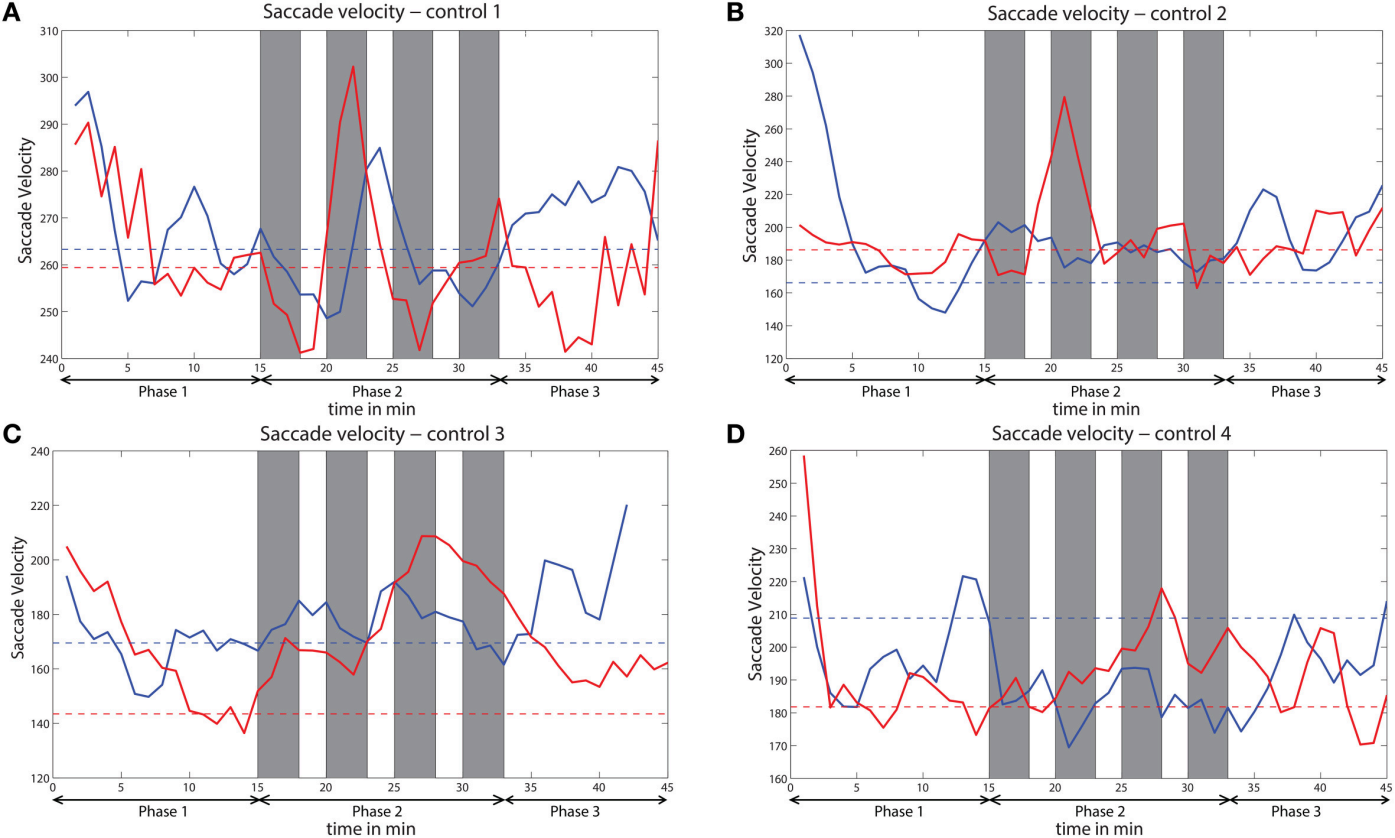
**Variation of saccade velocity across time for all the subjects (A–D) of the control study**. Solid red line indicates saccade velocity during challenge session and solid blue line indicates saccade velocity during no challenge session. Dashed red line indicates baseline for challenge session and dashed blue line indicates baseline for no challenge session. Gray bars indicate challenge stimulation periods.

The saccade velocity for the sessions with and without challenge integration was analyzed of all the four subjects who participated in the control study. Variation of saccade velocity of all the four subjects is presented in Figure 12. The saccade velocity of a subject during without challenge session is compared against saccade velocity during challenge session of that subject. The mean saccade velocity of the last 5 min of phase 1 is taken as the baseline in each session for each subject. The baselines of both the sessions are also shown. These baselines indicate the level of vigilance before the beginning of phase 2. For 3 out of 4 control subjects (controls 2, 3, and 4), the saccade velocity is relatively higher for the challenge session than the session without challenge during phase 2. However, for control 1 (Figure 9A), the variation of saccade velocity appears to be similar for both the sessions, except that the saccade velocity peaks are comparatively higher for the challenge session than for the session without challenge.

## 4. Discussion

### 4.1. Effect of challenge integration

We observed that eye movements and rhythmic oscillations in brain activity are modulated by challenging stimuli. From the eye tracking data, we found that challenging stimuli cause increasing peaks in saccade measures (saccade velocity and saccade amplitude) and suppression in blink rate (See Figure 3). Di Stasi et al. (2013) concluded that arousal leads to increase in saccade measures i.e., saccade amplitude and saccade velocity. Blink rate suppression in the case of interesting tasks is also observed in Yamada (1998). Hence, the reverse change in the variation of these measures due to challenging events suggest an increase in vigilance. Similarly, challenging stimuli caused suppression of relative delta power and augmentation in frontal midline theta and frontal theta to parietal alpha ratio in phase 2 showing an enhancement in the vigilance level. Delta power as investigated by Chuang et al. (2014) and Lal and Craig (2001) increases with increase in fatigue. Suppression of relative delta power due to challenge integration therefore suggests fatigue inhibition and increase in vigilance levels. Frontal midline theta also increases due to challenge integration indicating vigilance enhancement (Yamada, 1998). The increase in frontal theta to parietal alpha ratio suggests cortical arousal as studied by Smith and Gevins (2005) and Gevins et al. (1997). Moreover, the mean reaction time of phase 2 with integrated challenge (*RTmean* = 3.65 ± 1.4*s*) is found to be lower than phase 1 without challenge (*RTmean* = 4.6 ± 2.7*s*). Significant correlations between the EEG and eye tracking measures confirms the evidence of vigilance enhancement. The variation of saccade velocity of the subjects from control study are shown in Figure 12 for both challenge and no challenge sessions. Since saccade velocity is shown to decrease with vigilance decrement and vice versa, the results imply that the vigilance levels are higher for the sessions with challenge stimulation for 3 out of 4 subjects (controls 2, 3, and 4) during phase 2. Also, for the challenge session (indicated in red), the saccade velocity in phase 2 peaks higher than the indicated baseline (dashed red line) which suggests that the challenge stimulation leads to an increase in the vigilance levels of the subjects. This is consistent with the observations made in Figure 3 with respect to saccade velocity. However no such observations were made in the case of control 1. The reason for this behavior in control 1 may be due to subject to subject variation toward the challenge and needs further investigation.

### 4.2. Future directions

With respect to eye tracking data, blink suppression and increase in saccade velocity and amplitude are observed. As discussed in Di Stasi et al. (2013), it is interesting to note that saccade velocity increases as a function of amplitude, according to a relationship know as main sequence. This main sequence is found to be affected by time-on-task. However, the modulation of this relation due to the challenge integration has to be studied yet. In our task, the challenge integration results in a reverse change in delta power which suggests fatigue inhibition and improved attention. Increase in vigilance levels due to challenge integration suggests increased engagement of attentional resources due to increase in mental workload as supported by underload hypothesis of vigilance decrement. Fatigue and vigilance decrement mechanisms are compared and debated by several studies. Fatigue is believed to be an accumulated effect over time and can be ameliorated through breaks or rest during the tasks. However, vigilance decrement can be a result of either lack of motivation or increase of fatigue or both (Langner et al., 2012). It has been found that fatigue is accompanied by an increase in delta oscillations (Lal and Craig, 2001; Chuang et al., 2014). It could be that vigilance is a short-term state that can be altered by external stimulation while fatigue is a long-term state that can be temporarily altered but reverts back even though external stimulation can cause a transient adjustment in attention. This may be the reason why some subjects do not show a clear enhancement effect during challenge stimulation. Therefore, it might be necessary to inhibit fatigue in addition to providing external stimulation to improve sustained attention for performance enhancement. This aspect will be explored in detail in our future experiments where we plan to study the after effects of the challenge stimulation. The effect of challenge stimulation to sustain performance and inhibit increasing fatigue after the withdrawal of challenge has to be investigated further. The strategies of enhancing vigilance should also tend to increase the whole stimulus-response chain rather than simply enhancing perceptual sensibility of the stimulus. Hence in our future studies we plan to build a closed loop cognition enhancing strategies to take into account the enhancement of both stimulus perception and response to the stimulus.

## 5. Conclusion

In this study, we investigated the possibility of enhancing vigilance in a monotonous task using challenge integration. Challenge integration is achieved using noisy visual stimuli. The results obtained from EEG (relative delta power, frontal midline theta and frontal theta to parietal alpha ratio) and eye tracking measures (saccade velocity, saccade amplitude and blink rate), demonstrate with statistical significance, an increase in vigilance level due to challenge integration. Furthermore, we found strong correlations between EEG and eye tracking measures used to measure vigilance. Saccade velocity, saccade amplitude, frontal midline theta and frontal theta to parietal alpha ratio correlate positively while blink rate and relative delta power correlate negatively with vigilance levels. Therefore, challenge integration lead to increase in saccade velocity, saccade amplitude, frontal midline theta and frontal theta to parietal alpha ratio and suppression in blink rate and relative delta power. This study should find its application in fields like military surveillance, health monitoring and industrial watch-keeping where the primary task can be integrated with challenging stimuli to reduce vigilance decrement and enhance task performance.

## Author contributions

IB, HA, and NT have conceptualized the study and designed the experiments. IB performed the experiments. IB and JL performed the data analysis. All the authors made vital contributions in drafting the manuscript and have approved the final version.

## Funding

The authors thank the National University of Singapore for supporting the Cognitive Engineering Group at the Singapore Institute for Neurotechnology (SINAPSE) under grant R-719-001-102-232. This work was also partially supported by the Ministry of Education of Singapore under the grant MOE2014-T2-1-115.

### Conflict of interest statement

The authors declare that the research was conducted in the absence of any commercial or financial relationships that could be construed as a potential conflict of interest.
